# Peer Review of “Lessons Learned From the Resilience of Chinese Hospitals to the COVID-19 Pandemic: Scoping Review”

**DOI:** 10.2196/36685

**Published:** 2022-04-06

**Authors:** Ángela González-Santos

**Affiliations:** 1 Department of Physiotherapy Faculty of Health Sciences University of Granada Granada Spain

**Keywords:** COVID-19, pandemic, SARS-CoV-2, health care, hospitals, health care strategy, hospital resilience, interventions, crisis response, crisis preparedness, public health

*This is a peer-review report submitted for the paper “Lessons Learned From the Resilience of Chinese Hospitals to the COVID-19 Pandemic: A Scoping Review.”*

## Round 1 Review

### General Comments

1. This paper [1] is a scoping review with the aim to study the resilience during the COVID-19 pandemic of China’s hospitals during the first half of 2020.

### Specific Comments

#### Major Comments

- The background clearly presents the nature of the context, what is already known as well as the gap in knowledge and the need to conduct this study. The objectives of the scoping review are clear. The design is presented, and the authors follow the PRISMA-ScR (Preferred Reporting Items for Systematic Reviews and Meta-Analyses Extension for Scoping Reviews) Checklist.
- The characteristics of the sources of evidence and eligibility criteria are provided. The authors describe all information sources in the search and present the full search strategy for at least one database.
- Authors define the selection of sources of evidence, the data charting process, and all variables for which data were sought. They describe the methods used for conducting a critical appraisal of included sources of evidence, and the methods used for the synthesis of results.
- Results are correctly presented and cited in their manuscript section. The authors summarize the main results of the study, discuss the limitations of the scoping review, and provide a general conclusion of the outcomes.

#### Minor Comments

1. The authors refer to Table 4 three times in the manuscript; however, this table is not attached to the document. The same occurs with Table 3 that is referred to on page 7, second paragraph.
2. In relation to the PRISMA-ScR checklist, the eligibility criteria are not pointed out in the abstract section of the manuscript.
3. The registration number of the scoping review’s protocol is missing. The authors do not indicate if the protocol is available.
4. There are some typos in the manuscript, for example:
  - Authors use the acronym PPE (personal protective equipment) several times in the manuscript. Please indicate its meaning the first time it appears in the manuscript (page 10).
  - In the abstract section, the word “found” appears in a smaller size than the rest of the words.

## Abbreviations

PPE: personal protective equipment
PRISMA-ScR: Preferred Reporting Items for Systematic Reviews and Meta-Analyses Extension for Scoping Reviews
